# The role of noncoding RNA and its diagnostic potential in intrahepatic cholestasis of pregnancy: a research update

**DOI:** 10.3389/fgene.2023.1239693

**Published:** 2023-10-13

**Authors:** Liling Xiong, Mi Tang, Shasha Xing, Xiao Yang

**Affiliations:** ^1^ Obstetrics Department, Chengdu Women’s and Children’s Center Hospital, School of Medicine, University of Electronic Science and Technology of China, Chengdu, China; ^2^ GCP Institution, Chengdu Women’s and Children’s Center Hospital, School of Medicine, University of Electronic Science and Technology of China, Chengdu, China

**Keywords:** intrahepatic cholestasis of pregnancy, microRNA, lncRNA, circRNA, biomarker

## Abstract

Intrahepatic cholestasis of pregnancy (ICP) is a common liver disorder that generally occurs during the second or third trimester of pregnancy. It rarely causes any harm to the mother; however, it can result in short- and long-term complications in the offspring. Therefore, it is crucial to diagnose and treat this condition to avoid poor pregnancy outcomes. The identification of novel markers with potential diagnostic, prognostic, and therapeutic utility in ICP has gained attention. Noncoding RNAs (ncRNAs), including microRNA, long noncoding RNA, and circular RNA, are a type of transcripts that are not translated into proteins. They possess vital biological functions, including transcriptional and translational regulation and DNA, RNA, and protein interactions. The pathogenesis of ICP is related to the aberrant expression of several circulating or placenta-related ncRNAs. In this review, we summarized all recent findings on ncRNAs and ICP and outlined the concepts that form the basis for the early diagnosis and targeted treatment of ICP.

## 1 Introduction

Intrahepatic cholestasis of pregnancy (ICP) is a liver disease that particularly occurs during pregnancy. In general, maternal symptoms occur in the second or third trimester of pregnancy and primarily manifest as persistent pruritus and elevated levels of serum total bile acids (TBAs) and/or hepatic enzymes (Wood et al., 2018; Smith and Rood, 2020). Although ICP rarely causes any harm to the mother, it poses a significant risk to the fetus, manifesting as perinatal complications such as preterm birth, meconium-stained amniotic fluid, neonatal depression, respiratory distress syndrome, and stillbirth (Ozkan et al., 2015; Ovadia et al., 2019). Based on geography and demography, ICP incidence varies from 0.1% to 15.6% (X. X. Gao et al., 2020). At present, no effective treatment modalities are available for ICP, and the administration of ursodeoxycholic acid (UDCA) is a treatment option (Bicocca et al., 2018; Manna et al., 2019; Perez et al., 2006). The mechanism of action of UDCA is related to the displacement of hydrophobic bile acids to protect the hepatocyte membrane (Xia et al., 2018). Studies have reported that UDCA can relieve pruritus and decrease bile acid levels; however, whether it can prevent adverse effects on the fetus remains unproven (Chappell et al., 2019; Kumar and Kulkarni, 2020; Ovadia et al., 2021). At present, consensus on the diagnostic criteria for ICP is lacking. Nevertheless, most guidelines agree that pruritus and abnormal liver enzymes should be observed, with TBA levels being the most sensitive biochemical index for ICP diagnosis (Obstetrics Subgroup, 2015). The European Association for the Study of the Liver and the Society for Maternal–Fetal Medicine recommend persistent pruritus that resolves with delivery and bile acid levels of >10 μmol/L for diagnosis (Lee et al., 2021). Many prospective studies have reported that TBA levels of ≥40 μmol/L are associated with an increased risk of adverse neonatal outcomes in ICP (Glantz et al., 2004; Geenes et al., 2014; Kawakita et al., 2015). However, other studies have confirmed that TBA level alone is not a sufficiently sensitive and specific disease marker. Kondrackiene et al. conducted a retrospective study and reported that cholic acid (CA) and chenodeoxycholic acid (CDCA) levels and the CA/CDCA ratio are better disease markers (Brites et al., 1998; Kondrackiene and Kupcinskas, 2008). Accordingly, the identification of new markers with diagnostic and prognostic value in ICP is gaining attention.

The etiology and pathogenesis of ICP remain unclear. Nevertheless, several related theories about the reasons for ICP exist, including the estrogen–bile acid axis, placental hypoxia, immune disorders, trophoblast autophagy, and apoptosis (Ozkan et al., 2015; Xiao et al., 2021). However, genetic, epigenetic, and environmental factors may affect these pathogenic processes. For example, mutations in the protein-coding genes ABCB4 and ABCB11 may predispose women to intrahepatic cholestasis (Pauli-Magnus et al., 2004; Anzivino et al., 2013). Du et al. conducted a DNA microarray and revealed that the expression of immune-related genes gradually increased in the placentas of patients with mild and severe ICP; furthermore, histological experiments suggested that ICP is associated with immune activation (Du et al., 2014). In addition, DNA methylation and histone modification may play vital roles in ICP occurrence. A previous study has reported that prenatal CA exposure can elicit DNA methylation changes and result in later metabolic dysfunction in the offspring of ICP model mice (Papacleovoulou et al., 2013). Cabrerizo et al. have reported that the distal and proximal CpG sites of the FXR/NR1H4 promoter and the distal PXR/NR1I2 promoter have a lower methylation degree in patients with ICP (Cabrerizo et al., 2014). With respect to histone modifications, Shao et al. have reported that decreased HDAC3 expression in ICP may be associated with liver cell apoptosis; however, whether HDACs affect inflammatory cytokine expression should be explored further (Shao et al., 2017). Importantly, the importance of genetic and epigenetic regulation in ICP pathogenesis is gradually gaining appreciation.

In the human genome, although approximately 80% of the DNA can be transcribed into RNA, only a meager 1.5% of that RNA can be translated into proteins (Bhatti et al., 2021). Most RNA sequences that are not translated into proteins are called noncoding RNA (ncRNA). Because they cannot code proteins, ncRNAs were previously considered the evolutionary junk of the human genome; however, advances in next-generation sequencing technologies and bioinformatics analyses have helped in revealing the intricate roles of this RNA class in diverse biological functions, including pathway regulation and developmental and pathological processes. Based on their transcript size, ncRNAs are broadly categorized into two domains: ncRNAs with <200 nucleotides are called small ncRNAs, such as microRNAs (miRNAs), whereas those with >200 nucleotides are called long ncRNAs (lncRNAs) (Mercer et al., 2009). Furthermore, based on their expression and functional characteristics, ncRNAs can be divided into two types: housekeeping and regulatory ncRNAs (L. Gao et al., 2022). Housekeeping ncRNAs are fundamental and primarily expressed, whereas regulatory ncRNAs are synthesized at specific developmental phases or in response to some external stimuli (Romano et al., 2017). miRNAs, lncRNAs, and circular RNAs (circRNAs) are the most studied regulatory ncRNAs. ncRNAs play instrumental roles in RNA maturation, RNA processing, gene expression, and protein translation (Morris and Mattick, 2014; Palazzo and Lee, 2015; Anastasiadou et al., 2018).

In this review, we summarized the latest advances in the molecular mechanisms by which ncRNAs (i.e., microRNAs, lncRNAs, and circRNAs) contribute to ICP pathogenesis and reviewed the latest findings on their potential diagnostic utility as ICP biomarkers.

## 2 miRNAs and ICP

As small, endogenous ncRNAs, microRNAs regulate target messenger RNAs (mRNAs) by binding to a short complementary sequence that is often located in the 3′-untranslated region (UTR) of the mRNA. At present, most of the identified miRNAs are transcribed from introns; in contrast, a few miRNAs are intergenic, transcribed independently of a host gene, or less frequently, transcribed by the exons of protein-coding genes. The biogenesis of miRNAs begins in the nucleus. In the canonical pathway, RNA polymerase II/III transcribes primary miRNAs (pri-miRNAs) from their genes, followed by capping at the 5′ end and polyadenylation at the 3′ end. Thereafter, the microprocessor complex, which comprises the nuclear protein DiGeorge syndrome critical region 8 and Drosha, a ribonuclease III enzyme, processes the derived pri-miRNAs into double-stranded precursor miRNA (pre-miRNA). The exportin 5/RanGTP complex transports the pre-miRNAs to the cytoplasm, where their terminal loop is further cleaved by ribonuclease III Dicer, producing a double-stranded microRNA of approximately 22 nucleotides. The mature miRNA duplex can unwind and combine with argonaute2 to form a large ribonucleoprotein effector complex; this complex is called the RNA-inducing silencing complex, where the miRNAs repress or degrade the target mRNA in a sequence-specific manner. Using these mechanisms, miRNAs regulate >30% of the protein-coding genes in the human genome. One miRNA can regulate multiple mRNAs; on the other hand, multiple miRNAs can regulate one mRNA; this suggests that this tiny molecule has multiple and essential biological functions (Kabekkodu et al., 2018). During pregnancy, miRNAs are implicated in various processes, including the establishment of endometrial receptivity and regulation of the genes associated with immune responses, angiogenesis, and placental development (Bidarimath et al., 2014; Lycoudi et al., 2015). Changes in the expression of these molecules can result in abnormal placentation and pregnancy-related complications. To date, studies have reported that more than 40 miRNAs are differentially expressed in ICP tissues. The differentially expressed miRNAs involved in ICP pathogenesis are presented in Table 1.

**TABLE 1:** Dysregulation of miRNAs in ICP.

Study	Groups	Sample type	Stage of pregnancy	Targets	Functional pathway	miRNA
Kong et al. (2023)	14 ICP 14 CTRL	Plasma exosome	1st-3rd trimester and delivery	CDK16/PSEN1/JARID2	Embryonic development, cell proliferation	miRNA-940, miRNA-636, miRNA-767-3p (↑)
Zu et al. (2022)	46 ICP 46 CTRL	Serum	35th-41st week	—	Fatty acid biosynthesis, endoplasmic reticulum ubiquitin ligase complex, mTOR and AMPK signaling pathway	miRNA-7706, miRNA-877-3p, miRNA-128-3p, miRNA-1306-5p, and miRNA- 30c-5p (↑) miRNA-3613-5p, miRNA-379-5p, miRNA-4772-5p, and miRNA-204-5p (↓)
Dong et al. (2022)	60 ICP 48 CTRL	Serum exosome	3rd trimester	—	Lipid metabolism, apoptosis, oxidative stress, MAPK signaling pathway	miRNA-4271, miRNA-1275, miRNA-6891-5p (↓)
Feng et al. (2022)	10 ICP 10 CTRL	Placenta	34th-43rd week	PDCD4 and ESR1	Apoptosis	miRNA-21-5p (↓)
Wang et al. (2022)	7 ICP 5 CTRL	Placenta	term	KLRD1, BRAF, NFATC4	Signal transduction, immune system, endocrine system, cell growth and death	miRNA-7851-3p, miRNA-449a (↑) miRNA-372-3p, miRNA-371a-3p (↓)
Jiang et al. (2019)	82 ICP 64 CTRL	Urinary exosome	3rd trimester	ICAM1	Cell apoptosis and proliferation, angiogenesis and blood vessel regeneration	miRNA-21, miRNA-29a, miRNA-590-3p, miRNA-16, miRNA-584, miRNA-99b (↑) miRNA-151-3p, miRNA-200, miRNA-122, miRNA-26a-2, miRNA-520g (↓)
Zou et al. (2018)	40 ICP 40 CTRL	Serum	term	—	Lipid phosphorylation, apoptosis, MAPK signaling pathway	miRNA-371a-5p, miRNA-6865-5p, miRNA-1182 (↑)
Rao et al. (2017)	25 ICP 28 CTRL	Serum	term	PXR	PXR/MRP3 signaling pathway	miRNA-148a (↑)
(L. Ma et al., 2016)	40 ICP 50 CTRL	Urine	3rd trimester	—	TGF-β/TNF-α/IFN-γ pathway	miRNA-16, miRNA-671-3p, miRNA-369-5p (↑) miRNA-151-3p, miRNA-489, miRNA-300 (↓)
Zhang et al. (2014)	20 ICP 17 CTRL	Placenta and serum	36th-39th week	HLA-G	Immune response	miRNA-148a (↑)

### 2.1 miRNA pathways involved in ICP pathogenesis

#### 2.1.1 miRNAs in immune balance and estrogen regulation

Although the precise etiology of ICP remains unclear, immune imbalance and female hormones play vital roles in ICP occurrence and development (Diken et al., 2014; Larson et al., 2016); this finding is consistent with the fact that long-term oral contraceptive use or estrogen and progestin are high-risk factors for ICP development. Human leukocyte antigen G (HLA-G) is highly expressed in placental trophoblasts and plays a vital role in immune tolerance regulation in pregnancy and gestation maintenance (Xu et al., 2020). Zhang et al. have reported a marked decrease in the mRNA and protein expression of HLA-G in the placenta of patients with ICP (Zhang et al., 2014); however, they reported that miR-148a expression was markedly upregulated in the placenta; its levels were positively correlated with serum TBA levels in these patients. miR-148a upregulation in the placenta may inhibit HLA-G expression, thereby contributing to ICP pathogenesis. Consistent with the findings of this study, Rao et al. have reported that miR-148a expression was significantly upregulated in the serum of patients with ICP (Rao et al., 2017). Additional studies have reported that high estrogen levels in pregnant women with ICP can inhibit the mRNA and protein expression of pregnane X receptor (PXR) via miR-148a upregulation. Moreover, estrogen can upregulate the mRNA and protein expression of multidrug resistance protein 3 (MRP3). In conclusion, estrogen may act on the PXR/MRP3 signaling pathway by regulating miR-148a, thereby inducing ICP onset. Therefore, miR-148a may be related to ICP pathogenesis via immune balance and estrogen regulation.

#### 2.1.2 miRNAs and placental angiogenesis

A well-organized vascular structure comprises large vessels; however, the placentas of patients with ICP exhibit fewer and smaller blood vessels in each villus; this indicates that placental vascular function is impaired in a high-bile acid environment (Du et al., 2014; Voiosu et al., 2017). Previous studies have reported that bile acid may induce vasoconstriction in human fetal veins in the placental stem villus and umbilical vein (Gabzdyl and Schlaeger, 2015) and that the vasoconstriction of the umbilical vein may be responsible for fetal hypoxia, meconium inhalation, and even neonatal death in ICP (Ozkan et al., 2015). Vascular endothelial cell adhesion molecule-1 (VCAM-1) and intercellular adhesion molecule-1 (ICAM-1) belong to the immunoglobulin superfamily. They mediate leukocyte migration and adhesion, participate in angiogenesis, and promote trophoblast migration and proliferation (Singh et al., 2005; Schlesinger and Bendas, 2015). Qin et al. have reported that VCAM-1 expression is downregulated in placental vascular endothelial cells from patients with ICP (X. Qin et al., 2017). Furthermore, other studies have revealed that taurocholic acid (TCA) downregulates VCAM-1 expression via miR-590-3p and that the VCAM-1 gene participates in cell growth, apoptosis, and proliferation. In addition, in another study, the researchers analyzed cholestasis-related miRNAs and compared their expression in urinary exosomes isolated from patients with ICP. They observed that miR-21, miR-29a, and miR-590-3p were significantly upregulated in urinary exosomes isolated from patients with ICP compared with those isolated from healthy controls; furthermore, all three miRNAs can directly target and inhibit ICAM-1 expression (Jiang et al., 2019). Therefore, miR-21, miR-29a, and miR-590-3p may play significant roles in ICP pathogenesis.

#### 2.1.3 miRNAs and placental apoptosis

Several studies have reported that excessive bile acid can induce placental trophoblast apoptosis during ICP and affect placental function (H. Z. Wang et al., 2018; Xiao et al., 2021; Yang et al., 2022). However, the biological functions and regulatory mechanisms of microRNAs in placental apoptosis in patients with ICP remain unelucidated. Feng et al. have reported that miR-21-5p is significantly downregulated in ICP placental tissues and TCA-treated HTR8/SVneo cells (Feng et al., 2022). After TCA acts on HTR8/SVneo cells, miR-21-5p is downregulated, mediating the overexpression of the target genes PDCD4 and ESR1, thereby inducing trophoblast apoptosis. This finding suggests that miR-21-5p directly binds to the 3′-UTR of the mRNA of PDCD4 and ESR1 and inhibits the activation of the PDCD4 and ESR1 promoters. providing novel targets for ICP pathophysiology.

#### 2.1.4 miRNAs and lipid metabolism and mitogen-activated protein kinase (MAPK) and AMP-activated protein kinase (AMPK) signaling pathways

Recently, several studies on the serum miRNA profiles of patients with ICP have been published. Zou et al. have reported that miR-371a-5p, miR-6865-5p, and miR-1182 are significantly increased in the serum of patients with ICP compared with controls. Furthermore, bioinformatics analysis revealed that these three miRNAs principally affect lipid phosphorylation, apoptosis, and the MAPK signaling pathway (P. Zou et al., 2018). Consistent with their findings, Zu et al. measured differential miRNA expression in the peripheral blood samples of women with ICP and normal pregnant women and observed that 13 miRNAs were markedly increased in the serum of patients with ICP. Furthermore, bioinformatics analysis revealed that the differentially expressed miRNAs primarily affected lipid metabolism, the mammalian target of the rapamycin pathway, and the AMPK signaling pathway (Zu et al., 2022). In another study, the researchers extracted exosomes from the serum of normal pregnant women and those with ICP and observed that t miR-4217, miR-1275, and miR-6891-5p expression in maternal serum exosomes was significantly lower in patients with ICP than in controls. Bioinformatics analysis revealed that the three exosomal miRNAs were primarily associated with the MAPK signaling pathway, which is closely associated with lipid metabolism (Dong et al., 2022). In conclusion, the miRNAs in serum and serum exosomes may act on lipid metabolism and the MAPK and AMPK signaling pathways, thereby inducing ICP onset.

Taken together, the findings of the abovementioned studies suggest that the miRNAs in peripheral blood or placental tissues are vital for ICP development and progression. They can play a role in ICP occurrence and development by affecting immune balance, hormone regulation, apoptosis, lipid metabolism, and angiogenesis.

### 2.2 Diagnostic value of miRNAs in ICP

As an exclusionary diagnostic disease, ICP diagnosis primarily depends on clinical symptoms and TBA levels in the second or third trimester. However, TBA levels can vary with the fasting state or gestational age; therefore, novel prognostic and diagnostic biomarkers for ICP are urgently needed. Owing to their high stability in human fluids, miRNAs can be detected in different extracellular fluids, including urine, plasma, and serum (Mori et al., 2019; Ho et al., 2022). Therefore, molecular biology techniques can be used to measure miRNA expression to diagnose pregnancy-related diseases and guide the next therapeutic regimen via specific targets. To date, circulating miRNAs have been used as biomarkers for various pregnancy complications, including preeclampsia and gestational diabetes (Juchnicka et al., 2022; Kim et al., 2020; S. Qin et al., 2022). Several studies have elucidated the diagnostic value of ICP-related miRNAs by establishing receiver operating characteristic (ROC) curves. For example, Ma et al. conducted ROC analysis and compared the area under the ROC curves (AUCs) of the differentially expressed miRNAs in the urine of patients with ICP to determine their diagnostic values. They analyzed four miRNAs in combination. The AUC value of miR-151-3p, miR-671-3p, miR-369-5p, and miR-300 was 0.913, with sensitivity and specificity of 82.9% and 87%, respectively, for ICP diagnosis (L. Ma et al., 2016). Furthermore, the serum levels of these three miRNAs were significantly increased in patients with ICP, with AUC values of 0.771, 0.811, and 0.798 for miR-371a-5p, miR-6865-5p, and miR-1182, respectively. However, when these three miRNAs were combined, a more reliable ICP diagnosis was achieved, with the AUC value reaching 0.845. These three miRNAs are potentially associated with lipid metabolism, cell adhesion, cell aging, and the MAPK signaling pathway, as evidenced via bioinformatics analysis (P. Zou et al., 2018). Jiang et al. isolated urinary exosomes and observed that miR-21, miR-29a, and miR-590-3p expression was significantly upregulated in patients with ICP compared with normal pregnant women. The combination of the three miRNAs achieved a higher AUC value of 0.964; this indicates that these three exosomal miRNAs can be used together for ICP diagnosis (Jiang et al., 2019). In a recent study, the researchers identified that three miRNAs (miR-940, miR-636, and miR-767-3p) were upregulated in the plasma exosomes of patients with ICP. Additional analysis revealed that the AUC values of miR-940, miR-636, and miR-767-3p were 0.7591, 0.7727, and 0.8955, respectively (Kong et al., 2023). Taken together, these study findings suggest the higher diagnostic accuracy, sensitivity, and specificity of a combination of miRNAs. The study results of the value of miRNAs in ICP diagnosis are summarized in Table 2.

**TABLE 2:** Diagnostic value of miRNAs in ICP.

MiRNA	Sample type	Area under curve	Sensitivity	Specificity	Ref.
miR-151-3p/671-3p/369-5p/300	Urine	0.913	82.9%	87%	Ma et al. (2016)
miR-371a-5p	Serum	0.771	72.5%	75%	Zou et al. (2018)
miR-6865-5p	Serum	0.811	72.5%	81%	Zou et al. (2018)
miR-1182	Serum	0.798	75%	80%	Zou et al. (2018)
miR-29a	Urinary exosome	0.762	—	—	Jiang et al. (2019)
miR590-3p	Urinary exosome	0.753	—	—	Jiang et al. (2019)
miR-21	Urinary exosome	0.767	—	—	Jiang et al. (2019)
miR-16	Urinary exosome	0.750	—	—	Jiang et al. (2019)
miR-584	Urinary exosome	0.702	—	—	Jiang et al. (2019)
miR-99b	Urinary exosome	0.722	—	—	Jiang et al. (2019)
miR-4271	Serum exosome	0.861	93.3%	71.4%	Dong et al. (2022)
miR-1275	Serum exosome	0.886	96.7%	75%	Dong et al. (2022)
miR-6891-5p	Serum exosome	0.838	98.3%	72.9%	Dong et al. (2022)
miR-7706	Serum	1.00	74%	74%	Zu et al. (2022)
miR-877-3p	Serum	0.99	74%	77%	Zu et al. (2022)
miR-128-3p	Serum	0.97	72%	76%	Zu et al. (2022)
miR-940	Plasma exosome	0.759	63.64%	100%	Kong et al. (2023)
miR-636	Plasma exosome	0.773	72.73%	80%	Kong et al. (2023)
miR-767-3p	Plasma exosome	0.896	63.64%	100%	Kong et al. (2023)

## 3 lncRNAs and ICP

Structurally and functionally, lncRNAs represent an extremely diverse RNA group that differs from microRNAs. They are defined as transcripts of >200 nucleotides (Chan and Tay, 2018; Bridges et al., 2021). Based on the genomic loci of their origin, lncRNAs can be classified into six categories: sense, antisense, intergenic, intron, bidirectional, and enhancer lncRNAs (Moran et al., 2012). Similar to mRNAs, most lncRNAs are synthesized via RNA polymerase II activity (Dhanoa et al., 2018). lncRNAs can regulate biological processes via various mechanisms, including transcriptional and post-transcriptional gene expression regulation, chromatin modification, genomic imprinting, and other regulatory processes (Hombach and Kretz, 2016; Quinn and Chang, 2016). In particular, lncRNAs can activate or suppress gene expression by interacting with DNA, RNA, miRNAs, and proteins (Anfossi et al., 2018; Kaikkonen et al., 2011; Y. Ma et al., 2015). They can be combined with DNA or RNA molecules (such as mRNAs) via complementary base pairing (Q. Sun et al., 2018). Furthermore, lncRNAs interact with protein complexes or transcription factors to act as molecular scaffolds, affecting gene expression. lncRNAs crosstalk with miRNAs via molecular sponges and negatively regulate the functions of other RNA molecules by inhibiting miRNA effects; this phenomenon is called the competitive endogenous RNA (ceRNA) hypothesis (Guo et al., 2021; Su et al., 2021; H. Zhang et al., 2021). Although lncRNAs were defined as transcripts without coding ability for a long time, the development of bioinformatics has increased the evidence of the presence of small open reading frames (ORFs) within lncRNAs (Chugunova et al., 2018). These ORFs may bind to ribosome-associated proteins, thereby suggesting that lncRNAs contain translated coding regions that encode peptides (Nam et al., 2016; Guerra-Almeida et al., 2021; Vitorino et al., 2021). This finding questions the noncoding nature of lncRNAs.

Several studies have applied transcriptomics analysis to identify the lncRNAs involved in ICP. To date, studies have described the specific roles of several lncRNAs in ICP development and progression. The upregulation and downregulation of specific lncRNAs can affect critical mechanisms underlying ICP development and lead to changes in trophoblast proliferation, invasion, and autophagy. Table 3 summarizes the differentially expressed lncRNAs that participate in ICP pathogenesis.

**TABLE 3:** Dysregulation of specific lncRNAs and proposed mechanisms of action in ICP.

lncRNA	Differential expression	Samples	Targets	Functional pathway
H19	Upregulation	Cholangiocyte	SHP	Upregulation of lncRNA H19 in cholangiocyte can be delivered to hepatocyte and suppress SHP expression by inhibiting promotor activity and reducing mRNA stability, which induced an excessive inflammatory response. Li et al. (2018)
Linc02527	Upregulation	Placenta and serum	miRNA-3185/YBX1	The linc02527 upregulation promotes trophoblast autophagy through sponging miR-3185 or directly binding to YBX1 and activated P21. Hu et al. (2018)
ASO3480	Downregulation	Serum	—	Lipid metabolism, apoptosis, cell cycle, cell differentiation and oxidative stress. Zou et al. (2021)
ENST00000505175.1	Downregulation	Serum	Oct-1	Modulate cell proliferation, migration and invasion by regulating ERK and other signaling pathways. Zou et al. (2021)
ENST00000449605.1	Downregulation	Serum	—	Lipid metabolism, apoptosis, cell cycle, cell differentiation and oxidative stress. Zou et al. (2021)

### 3.1 lncRNA pathways involved in ICP pathogenesis

#### 3.1.1 Estrogen enhances the role of the lncRNA H19 in cholestasis

H19 is a maternally expressed lncRNA with imprinting properties as well as a target gene of estrogen. It plays an essential role in normal embryonic development. Using a mouse model, Li et al. (2018) observed that H19 expression in the bile ducts of mice with cholestatic liver disease differed based on sex and that H19 expression was higher in multidrug resistance gene 2 knockout (Mdr2^−/−^) female mice with severe cholestasis than in age-matched Mdr2^−/−^ male mice. In addition, they injected H19 short hairpin RNA adenovirus into the tail vein of Mdr2^−/−^ female mice to decrease H19 expression. After injection, alkaline phosphatase and bile acid levels were significantly decreased in female mice. The low H19 expression significantly decreased cholestatic injury in female Mdr2^−/−^ mice. These findings suggest that H19 plays a vital role in cholestatic disease and that estrogen may enhance its role in intrahepatic cholestasis in mice, a key factor responsible for the sex difference in liver function impairment in mice. During pregnancy, the body is in a high estrogenic state, and H19 may be involved in ICP occurrence and development; however, the specific mechanism of action remains unelucidated.

#### 3.1.2 linc02527 promotes autophagy in ICP

linc02527 is a lncRNA that has been implicated in liver cancer and other liver diseases. Hu et al. have reported that linc02527 is higher in the serum and placental tissues of patients with ICP than in those of normal pregnant women. Furthermore, serum TBA, glycine, alanine aminotransferase, and aspartate aminotransferase levels are positively correlated with linc02527 expression in placental tissues (Hu et al., 2018). In addition, the HTR8/SVneo cell experiment revealed that autophagy improved in cells infected with the linc02527 lentivirus expression vector and that miR-1323, miR-3185, miR-3935, miR-3187, miR 3663, and miR-515 are significantly downregulated. Moreover, the luciferase reporter gene assay confirmed the binding of miR-3185 to linc02527, and miR-3185 overexpression suppressed linc02527 expression. Hu et al. further improved the ICP mouse model and observed that when autophagy was activated, the growth of fetal mice was inhibited; in contrast, chloroquine-induced autophagy inhibition improved the growth of fetal mice. Taken together, these study findings suggest that linc02527 is a potential biomarker for ICP during pregnancy and can be a potential therapeutic target for fetal growth restriction in patients with ICP.

#### 3.1.3 Serum lncRNAs and fatty acid metabolism, cell cycle, apoptotic signaling, and the oxidative stress pathway

Zou et al. elucidated the expression profiles of lncRNAs by collecting the serum samples of patients with ICP and explored the diagnostic and prognostic values of the differentially expressed lncRNAs in ICP. They revealed that 58 lncRNAs were upregulated and 85 were downregulated in patients with ICP compared with healthy controls (S. Zou et al., 2021). Furthermore, to verify this finding, the expression of three lncRNAs, namely, ENST00000449605.1, ASO3480, and ENST00000505175.1, was measured. Their expression was significantly decreased and exhibited diagnostic and prognostic values in ICP. In addition, bioinformatics analysis revealed that fatty acid metabolism, the apoptotic signaling pathway, cell cycle regulation, and oxidative stress were the primarily enriched target pathways.

### 3.2 Diagnostic value of lncRNAs in ICP

Studies on the biological functions and roles of lncRNAs as potential ICP biomarkers are limited. Zou et al. investigated the diagnostic and prognostic values of ENST00000449605.1, ASO3480, and ENST00000505175.1 in ICP, established ROC curves, and computed the AUCs values, which were 0.812, 0.798, and 0.731, respectively (S. Zou et al., 2021). When the three lncRNAs were combined, the AUC value increased to 0.865; this finding indicates that the combined use of these three lncRNAs is a more reliable diagnostic marker for ICP. Furthermore, they performed the chi-squared test to determine the prognostic value of these three lncRNAs and observed low lncRNA expression is associated with worse perinatal outcomes, including meconium-stained amniotic fluid, fetal distress, and premature delivery; this finding indicates that these three lncRNAs can be negative prognostic factors for the risk of ICP. Additional experiments revealed that ENST00000505175.1 can target Oct-1 and modulate cell proliferation, migration, and invasion by regulating the ERK signaling pathway, possibly playing a role in ICP pathogenesis.

## 4 circRNAs and ICP

circRNAs are produced via back splicing, a process in which the 3′ end of a downstream exon is spliced and covalently linked with the 5′ end of an upstream exon. Based on their source, circRNAs can be divided into three categories: intron (circular intronic RNA), exon (exonic circRNA), and both exon and intron (exon‒intron circular RNA). Because they lack a 5′ terminus with a cap and a 3′ terminus with a poly(A) tail, circRNAs are resistant to degradation via nucleic acid exonucleases. These characteristics allow circRNAs to fulfill many biological functions, including gene transcriptional regulation, miRNA molecular sponging, RNA-binding protein interaction, and protein translation.

Studies on the role of circRNAs in ICP remain limited. However, some studies have reported that circRNAs may be involved in ICP development and progression. In a study, circ0060731 expression was measured in the placenta samples of 10 normal and 10 pregnant women with ICP. circ0060731 expression was significantly higher in the women with ICP than in normal women (Feng et al., 2022). Additional experiments revealed that circ0060731 can downregulate miR-21-5p and that miR-21-5p can directly bind to the mRNAs of PDCD4 and ESR1 and inhibits the activation of the PDCD4 and ESR1 promoters; this results in the apoptosis of placental trophoblasts in ICP. Wang et al. collected placental tissues from pregnant women with ICP and normal pregnant women for whole-transcriptome sequencing and measured circRNA expression in the placentas of the women in these two groups. They identified 157 differentially expressed circRNAs, including 91 upregulated and 66 downregulated circRNAs, in the ICP group. These differentially expressed circRNAs may mediate immune dysfunction in the placentas of patients with ICP (Y. Wang et al., 2022). Taken together, these findings suggest that circRNAs play a role in the molecular mechanisms underlying ICP. Nevertheless, additional studies are warranted to better understand the specific functions of these circRNAs and their potential diagnostic or therapeutic implications for ICP.

## 5 ceRNA regulatory network in ICP

The ceRNA hypothesis proposes that RNA transcripts, both coding and noncoding, crosstalk with and regulate each other via microRNA response elements (Thomson and Dinger, 2016). The ceRNA hypothesis suggests that the regulatory interactions between different RNA types are important for the development, progression, and regulation of various diseases, including cancer, cardiovascular diseases, and neurological disorders (Cai et al., 2022; Liang et al., 2020; X. Wang et al., 2021). Furthermore, increasing evidence suggests that ceRNAs play a vital role in reproductive health, including fetal development, preeclampsia, gestational diabetes, and spontaneous abortion (Gan et al., 2022; Jain et al., 2022; Leng et al., 2019; S. Liu et al., 2019).

In ICP, the ceRNA regulatory network is disrupted, leading to dysregulated gene expression and disease development. Several studies have identified the key genes and pathways that are dysregulated in the ceRNA regulatory network in ICP. For example, circ0060731 modulates miR-21-5p expression in the placentas of patients with ICP, leading to the upregulation of its target genes, including PDCD4 and ESR1; this, in turn, results in the impaired function of placental trophoblasts (Feng et al., 2022). Furthermore, linc02527 regulates ATG5 and ATG7 by sponging miR-3185, leading to the dysregulation of the autophagy signaling pathway, which plays a role in ICP pathogenesis (Hu et al., 2018). Other genes and pathways that are dysregulated in the ceRNA regulatory network in ICP include the Wnt signaling pathway, which plays a role in liver regeneration and fibrosis; the IL-17 signaling pathway, which plays a role in inflammation and immune responses; and the Hippo signaling pathway, which regulates cell proliferation and apoptosis (Fang et al., 2022; Y. Wang et al., 2022).

Collectively, the dysregulation of the ceRNA regulatory network is a vital factor for ICP development. Nevertheless, additional studies are warranted to fully understand the mechanisms underlying this dysregulation and to identify potential therapeutic targets for intervention.

## 6 Conclusion and future directions

**FIGURE 1:**
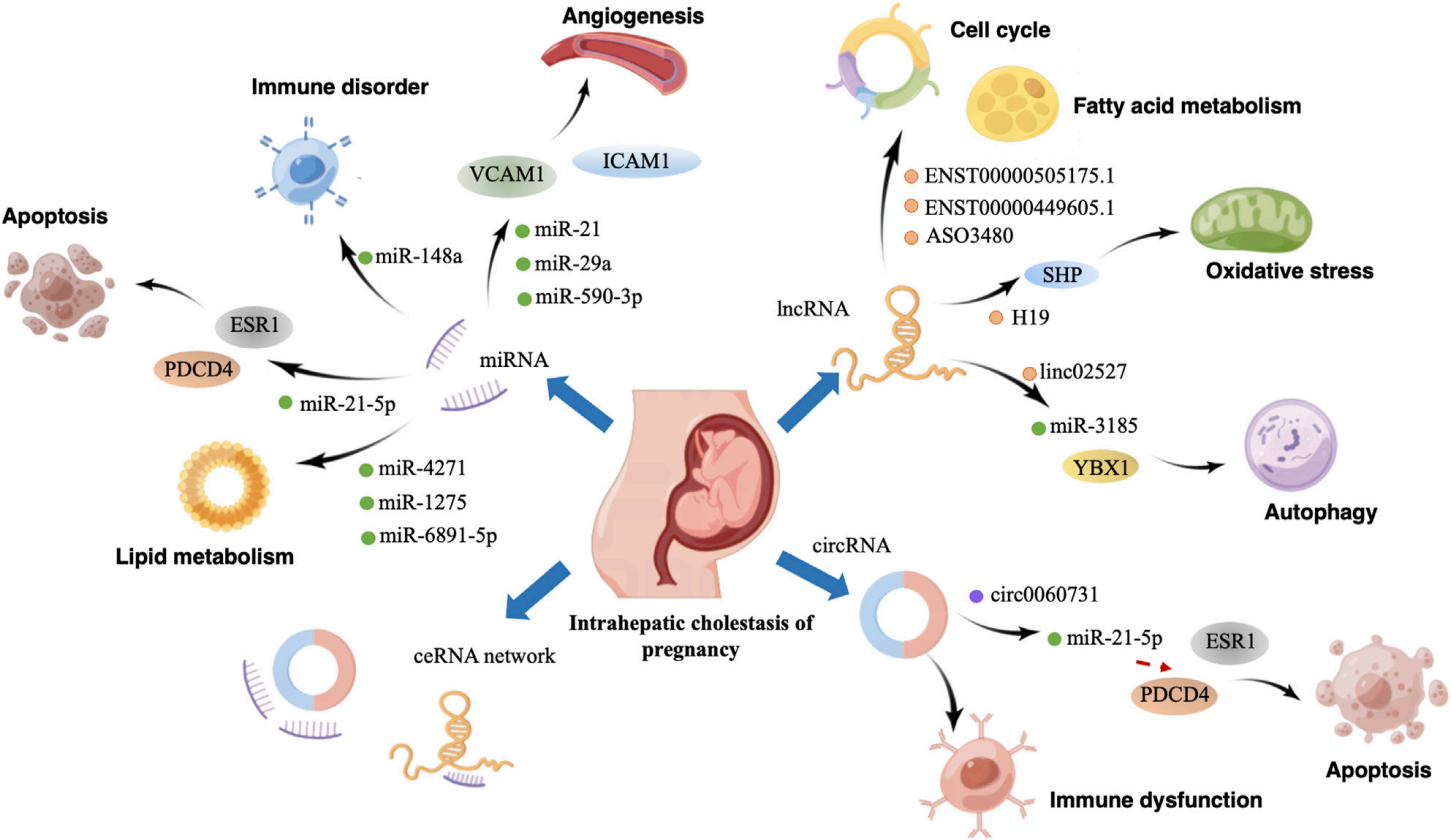
Graphical abstract. Effect and mechanism of non-coding RNA in the pathogenesis of intrahepatic cholestasis of pregnancy. By Figdraw.

ICP is characterized by extensive placental dysfunction caused by immune disorders, autophagy, trophoblast apoptosis, and lipid metabolism dysregulation (Mathur et al., 2022; Shan et al., 2021; X. Sun et al., 2022). With continuous advances in molecular biology, studies on ICP have gradually reached the genetic level. Several studies have indicated that ncRNA dysregulation can impair trophoblast function and subsequently lead to ICP development. Furthermore, the upregulation and downregulation of specific miRNAs and lncRNAs can function as biomarkers for ICP diagnosis and prognosis. Therefore, understanding the role of ncRNAs and their diagnostic potential in ICP is vital for clinical applications. Figure 1 more intuitively shows the role of ncRNA in the pathogenesis of ICP.

Although there are studies on the roles and mechanisms of ncRNAs in ICP, they have several limitations. First, in recent molecular biology studies on ICP, quantitative real-time reverse transcription polymerase chain reaction was widely used. These studies identified many ncRNAs with abnormal expression. However, this method can only detect the expression of known genes and fails to directly determine gene sequences or discover unknown sequences; furthermore, studies on differentially expressed ncRNAs are not comprehensive. Second, most studies on ncRNAs and ICP have focused on specific ncRNAs, such as microRNAs. Therefore, accurately interpreting the role of ncRNAs in ICP and other diseases remains challenging. Extensive studies on the expression and functions of all ncRNAs in ICP are warranted. Third, many studies on ncRNA and ICP have been conducted with a small number of patients, limiting the generalizability of the findings. Large-scale studies are warranted to confirm the roles of ncRNAs in ICP. Finally, at present, ICP diagnosis is based on clinical symptoms and biochemical tests. However, standardized criteria for ICP diagnosis are lacking, resulting in differences in patient selection and making it challenging to compare results across studies.

Despite these limitations, there is considerable hope for future studies on ncRNAs and ICP. Over the past decade, noncoding RNA-based therapeutics have exhibited considerable potential for developing targeted therapies for several diseases (Matsui and Corey, 2017; Brichant et al., 2021). Using RNA interference, both miRNA inhibitors and mimics can regulate the expression of targeted genes and mRNAs with high specificity, thereby representing a remarkable targeted therapeutic tool for ICP treatment. At present, oligonucleotide-based drugs targeting or mimicking ncRNAs are being developed against various targets and tested in clinical trials (van der Ree et al., 2016; Matsui and Corey, 2017). For example, Gomez et al. have developed single-stranded oligonucleotides complementary to miR-21 and reported that they can be used to treat Alport nephropathy (Gomez et al., 2015). However, to successfully apply ncRNA therapeutics in clinical settings, safe and effective nanodelivery systems are warranted; therefore, promising nanodelivery/nanoparticle-based approaches with decreased side effects should be developed in the future. On the other hand, our understanding of their mechanisms will improve as technologies and methods to study ncRNAs are continuously developed. Recent developments in high-throughput technologies, including mass spectrometry, microarray, and sequencing technologies, have facilitated the analysis of the ncRNA expression profiles of many diseases. Real-time polymerase chain reaction and the more advanced droplet digital polymerase chain reaction can be used for experimental validation (Qian et al., 2019). Moreover, single-cell RNA sequencing (scRNA-seq) facilitates the in-depth analysis of differentially expressed ncRNAs (Jovic et al., 2022), and the scRNA-seq technique has favourable potential in the study of ICP because it is a powerful tool for characterizing individual cells and elucidating biological mechanisms at the cellular level (Y. Liu et al., 2018; T. Zhang et al., 2021). Furthermore, studies on ncRNAs in ICP will likely benefit from integrating knowledge from other fields, including genetics, epigenetics, and environmental factors. This interdisciplinary approach will facilitate a more comprehensive understanding of the disease and its underlying mechanisms.

In this review, we summarized the discovered ncRNAs with important biological functions in ICP and provided a novel perspective for the early diagnosis and targeted treatment of ICP. Advances in high-throughput sequencing and other advanced technologies have made the study of complete ncRNA transcriptome possible. Furthermore, the pathogenesis and characteristics of ICP can be understood more comprehensively via ncRNAs. In addition, relevant gene targets can be identified for early prediction, diagnosis, and treatment, which is conducive to the improvement and optimization of ICP diagnosis and treatment and to decrease adverse pregnancy outcomes.

## Figures and Tables

**Table:** 

**ICP**	Intrahepatic cholestasis of pregnancy
**ncRNA**	noncoding RNA
**mRNA**	messenger RNA
**miRNA**	microRNA
**lncRNA**	long noncoding RNA
**circRNA**	circular RNA
**TBAs**	total bile acids
**UDCA**	ursodeoxycholic acid
**EASL**	The European Association for the Study of the Liver
**SMFM**	the Society for Maternal-Fetal Medicine
**CA**	cholic acid
**CDCA**	chenodeoxycholic acid
**pri-miRNA**	primary miRNA
**pre-miRNA**	precursor miRNA
**DGCR8**	DiGeorge Syndrome Critical Region 8
**Ago2**	Argonaute2
**RISC**	RNA-inducing silencing complex
**ceRNA**	competitive endogenous RNA
**ORF**	open reading frame
**RBP**	RNA binding protein
**HLA-G**	human leukocyte antigen G
**PXR**	pregnane X receptor
**MRP3**	multidrug resistance protein 3
**VCAM-1**	vascular endothelial cell adhesion molecule-1
**ICAM-1**	intercellular adhesion molecule-1
**TCA**	taurocholic acid
**mTOR**	mammalian target of rapamycin
**AMPK**	AMP-activated protein kinase
**MAPK**	mitogen activated protein kinase
**ROC**	receiver operating characteristic
**shRNA**	short hairpin RNA
**ALP**	alkaline phosphatase
**ALT**	alanine aminotransferase
**AST**	aspartate aminotransferase
**MRE**	microRNA response element
**scRNA-seq**	single-cell RNA sequencing
